# KLF5 regulates epithelial-mesenchymal transition of liver cancer cells in the context of p53 loss through miR-192 targeting of ZEB2

**DOI:** 10.1080/19336918.2020.1826216

**Published:** 2020-10-07

**Authors:** Lan Sun, Xiaona Zhou, Yanmeng Li, Wei Chen, Shanna Wu, Bei Zhang, Jingyi Yao, Anjian Xu

**Affiliations:** a Department of Pathology, Beijing Friendship Hospital, Capital Medical University, Beijing, China; b Department of General Surgery, Beijing Friendship Hospital, Capital Medical University, Beijing, China; c Experimental Center, Beijing Friendship Hospital, Capital Medical University, Beijing, China; d National Clinical Research Center for Digestive Disease, Beijing Friendship Hospital, Capital Medical University, Beijing, China; e Clinical Laboratory Center, Beijing Friendship Hospital, Capital Medical University, Beijing, China

**Keywords:** KLF5, miR-192, epithelial-mesenchymal transition, liver cancer, ZEB2

## Abstract

Krüppel-like factor 5 (KLF5) can both promote and suppress cell migration, but the underlying mechanisms have not been elucidated. In this study, we show that the function of KLF5 in epithelial-mesenchymal transition (EMT) and migration of liver cancer cells depends on the status of the cellular tumor antigen p53 (p53). Furthermore, zinc finger E-box-binding homeobox 2 (ZEB2) is the main regulator of KLF5 in EMT in liver cancer cells in the context of p53 loss. Most importantly, the regulation of ZEB2 by p53 and KLF5 is indirect and that miR-192 mediates this regulation. Finally, we find that in invasive liver cancer, KLF5 is absent in the context of p53 loss or mutation.

## Introduction

Krüppel-like factor 5 (KLF5, IKLF5 or BTEB2) is a basic transcriptional factor that is ubiquitously expressed in different tissues [1]. Multiple cellular processes, including cell proliferation, cell apoptosis, differentiation and migration, are mediated or regulated by KLF5 [2]. However, many studies have reported opposing functions of KLF5 in the same cellular process in different cell types [3–5]. For example, KLF5 can be both anti- and pro-tumorigenic in prostate cancer cells [6–8], and it can promote proliferation of primary esophageal keratinocytes but inhibit growth of esophageal cancer cells [5]. In terms of cell migration, KLF5 can promote keratinocyte migration by inducing integrin-linked kinase and can promote bladder cancer cell and breast cancer cell migration by upregulating the tyrosine-protein kinase Fyn (FYN) and TNF alpha-induced protein 2 (TNFAIP2), respectively [9–11]; in contrast, KLF5 loss can also drive the invasive progression of human squamous cell cancers in the context of p53 ablation [12], and epithelial cell migration is accelerated after KLF5 knock-down [13]. Thus, the intrinsic relationship between migration and KLF5 remains to be elucidated.

Cell migration is often associated with epithelial-mesenchymal transition (EMT) during normal development and cancer progression [14,15]. The phenotypic changes in EMT include loss of cell-cell adhesion mediated, which is by E-cadherin downregulation and vimentin upregulation, acquisition of motility, and expression of several EMT activators (such as Snail, Slug, Twist, ZEB1 and ZEB2) [16,17]. Although several studies have reported the inhibitory function of KLF5 in EMT [18,19], this effect is not always observed. For example, in esophageal keratinocytes with wild type p53, KLF5 suppression cannot induce EMT [12]. Therefore, the role of KLF5 in EMT could also be context-dependent, b the mechanisms involved are still unknown.

In this study, we first investigated the role of KLF5 in EMT and the migration of liver cancer cells. Interestingly, we found that the function of KLF5 in EMT and migration of liver cancer cells depended on the p53 status. Specifically, KLF5 inhibited EMT in liver cancer cells and inhibited cell migration only when p53 function was lost. Next, we clarified the role of KLF5 in EMT in liver cancer cells in the context of p53 loss. ZEB2, an important activator of EMT, was found to be the main regulator of KLF5 in EMT liver cancer cells in the context of p53 loss. Furthermore, we found that KLF5 and p53 coordinately regulated the expression of miR-192, which repressed EMT by targeting ZEB2. Finally, we found that in invasive liver cancer, KLF5 was absent in the context of p53 loss or mutation. Therefore, KLF5 loss could be a valuable diagnostic target for invasive liver cancer when p53 is lost or mutated.

## Results

### Function of KLF5 in EMT in liver cancer cells depends on p53 status

**Figure 1.**
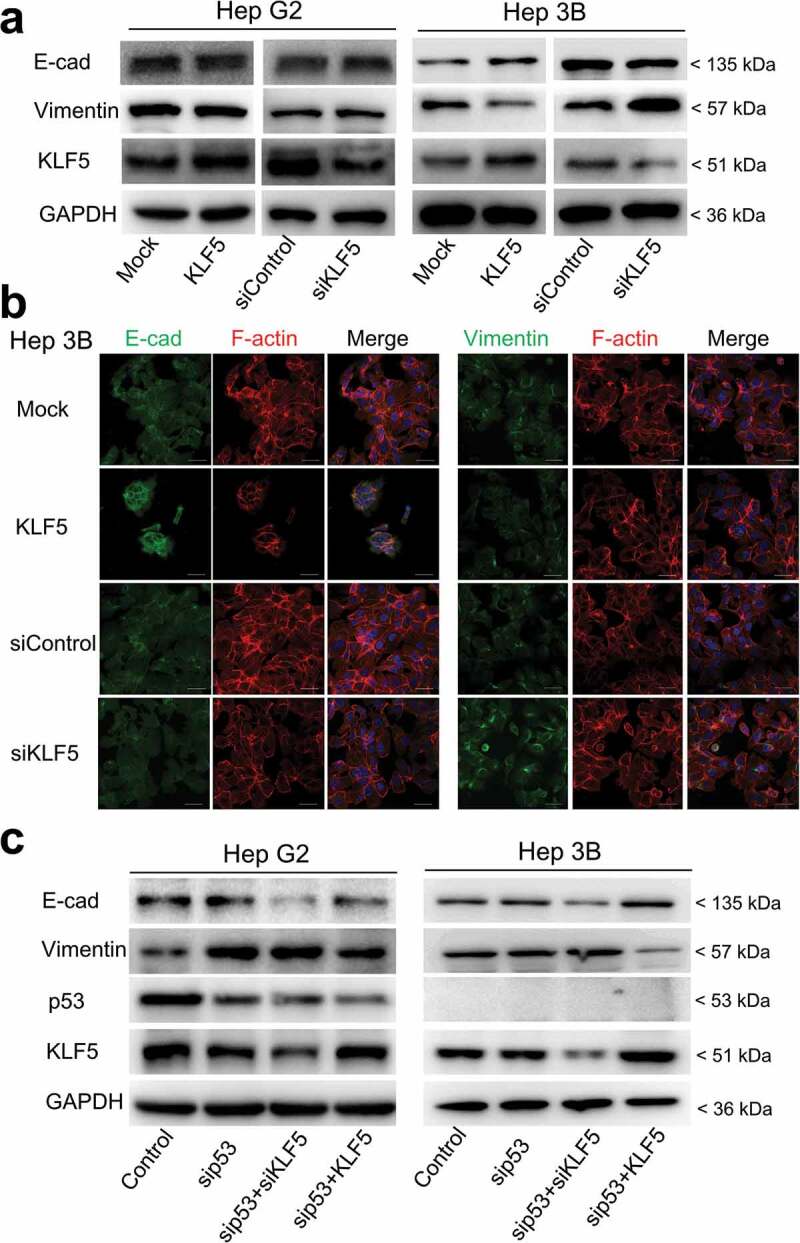
KLF5 regulates EMT marker expression in the context of p53 loss. (a). The expression levels of the EMT markers E-cadherin and vimentin were regulated by KLF5 in the p53-null cell line Hep3B (right), while in p53 wild type HepG2 cells, the expression levels of E-cadherin and vimentin were not altered (left). (b). Changes in the expression levels of E-cadherin and vimentin were examined by immunofluorescence staining in Hep3B cells after KLF5 overexpression or knock-down. F-actin (actin cytoskeleton) staining was used to show the cell morphology. Scale bar = 50 µm. (c). KLF5 expression altered the E-cadherin and vimentin expression levels in HepG2 cells after p53 silencing and in p53-null Hep3B cells, which suggests that the p53 status influenced the function of KLF5 in liver cancer cell EMT.

To investigate the function of KLF5 on EMT in liver cancer cells, we overexpressed or knocked down KLF5 in the liver cancer cell lines HepG2 and Hep3B. Surprisingly, these two liver cancer cell lines presented different results. In HepG2 cells, KLF5 exhibited no effect on EMT-related protein expression. Overexpression and knock-down of KLF5 did not alter the expression of E-cadherin and vimentin proteins. In contrast, KLF5 acted as an EMT inhibitory factor in Hep3B cells, as E-cadherin expression was increased and vimentin expression was decreased in KLF5-overexpressing cells, and E-cadherin expression was decreased and vimentin expression was increased in KLF5 knock-down cells (Figure 1(a) and Fig. S1). Furthermore, we detected the expression of E-cadherin and vimentin by immunofluorescence staining of Hep3B cells after KLF5 overexpression or knock-down. Consistent with the results of western blotting, the expression of E-cadherin was increased, while the expression of vimentin was decreased in KLF5-overexpressing cells. On the contrary, in KLF5 knock-down cells, the expression of E-cadherin was decreased, while the expression of vimentin was increased (Figure 1(b)).

To explore the reason behind these findings, we noted that these two cell lines had different p53 statuses: HepG2 cells harbor wild type p53, while Hep3B is a p53-null cell line. To confirm whether KLF5 function in liver cancer cell EMT is dependent on p53 status, we knocked down p53 and then overexpressed or knocked down KLF5 in HepG2 and Hep3B cells. As shown in Figure 1(c), consistent with a previous report, the knock-down of p53 promoted EMT, as decreased expression of E-cadherin and increased expression of vimentin were observed. Notably, when p53 was knocked down, KLF5 knock-down further decreased the expression of E-cadherin, while overexpression of KLF5 decreased the expression of vimentin. These results indicated that p53 status influenced the EMT inhibitory function of KLF5 in liver cancer cells.

### P53 status determines the effect of KLF5 on liver cancer cell migration

**Figure 2.**
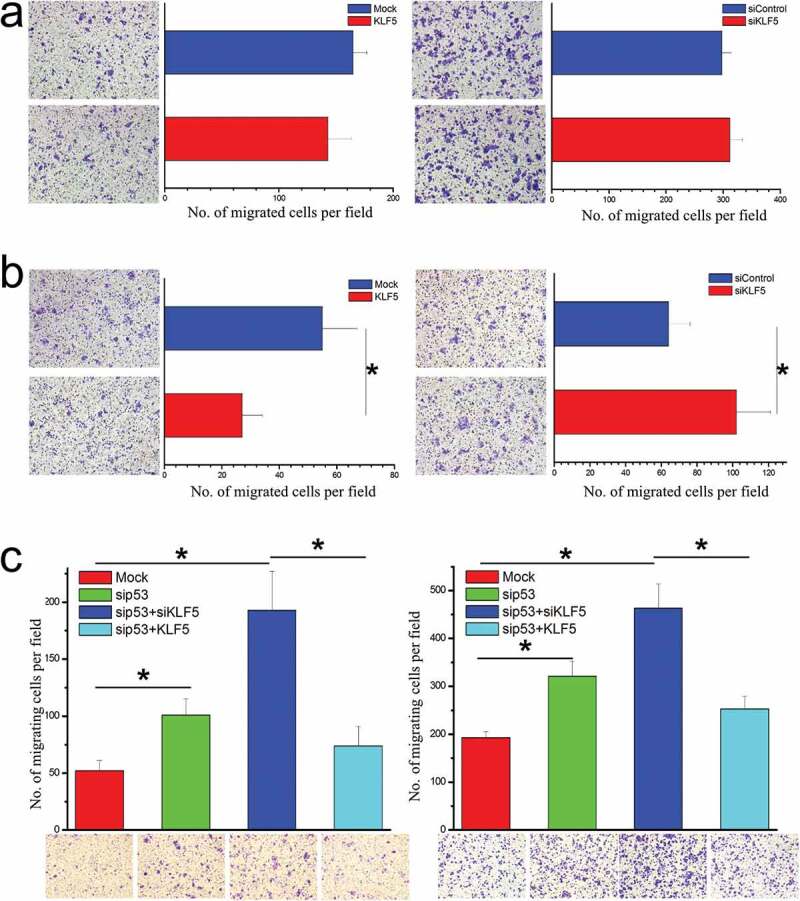
P53 status determines the regulation of cell migration by KLF5. (a). In HepG2 cells harboring wild type p53, KLF5 overexpression (left) and knock-down (right) had little effect on cell migration. (b). In p53-null Hep3B cells, KLF5 overexpression significantly suppressed cell migration (left), while KLF5 knock-down significantly promoted cell migration (right). (c). In the p53 wild type cell lines HepG2 (left) and BEL-7402 (right), KLF5 significantly influenced cell migration after p53 silencing. The number of migrating cells was counted in 4 random fields (n = 4 from 3 independent experiments). *P < 0.05, data are presented as the means ± SD.

Since EMT is closely related to cell migration, we next questioned whether p53 status affected the regulation of cell migration by KLF5. As shown in Figure 2(a), KLF5 did not influence the migration of HepG2 cells harboring wild type p53. Furthermore, we confirmed the results in another p53 wild type liver cancer cell line BEL-7402 (Fig. S2) and found that migration of p53-null Hep3B cells was significantly suppressed by KLF5 (Figure 2(b)). Notably, KLF5 exhibited a significant cell migration-inhibitory effect in the context of p53 knock-down in both HepG2 and BEL-7402 cells (Figure 2(c)), which confirmed that p53 status determined the effect of KLF5 on liver cancer cell migration, possibly through EMT regulation.

### P53-dependent effect of KLF5 on EMT in liver cancer cells via ZEB2 regulation

**Figure 3.**
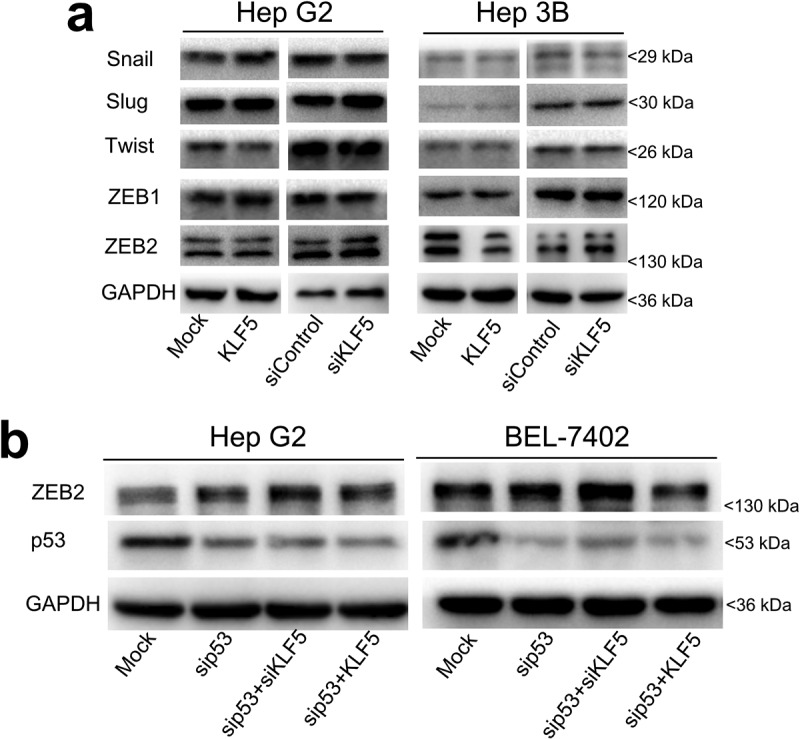
EMT activator ZEB2 mediates the regulation of EMT by KLF5. (a). The protein expression levels of EMT activators, including snail, slug, twist, ZEB1 and ZEB2, were examined in HepG2 (left) and Hep3B (right) cells after KLF5 overexpression and knock-down. Notably, only ZEB2 protein expression was decreased in Hep3B cells after KLF5 overexpression and was increased after KLF5 knock-down, which suggests that ZEB2 could be the primary factor involved in the regulation of EMT by KLF5 in the context of p53 loss. (b). The regulation of ZEB2 expression by KLF5 in the context of p53 loss was confirmed by p53 silencing in the p53 wild type cell lines HepG2 and BEL-7402, which suggests that the regulation of ZEB2 by KLF5 was dependent on the p53 status.

To identify EMT regulators that are involved in the p53-dependent effect of KLF5 on liver cancer cell EMT, we first tested the expression levels of the known EMT activators Snail, Slug, Twist, ZEB1 and ZEB2 in HepG2 and Hep3B cells after KLF5 overexpression and knock-down. Our results show that the expression of Snail, Slug, Twist and ZEB1 was not significantly altered in these two cell lines after KLF5 overexpression and knock-down. However, although the mRNA expression of ZEB2 was not significantly altered (Fig. S3), the protein expression of ZEB2 was decreased in p53-null Hep3B cells after KLF5 overexpression and was increased after KLF5 knock-down. However, the protein expression of ZEB2 was not obviously different after KLF5 overexpression or knock-down in the p53 wild type cell line HepG2 (Figure 3(a)). To confirm the regulatory effect of KLF5 on ZEB2 in the context of p53 loss, we knocked down p53, then overexpressed or knocked down KLF5 in HepG2 and BEL-7402 cells and examined ZEB2 expression. As shown in Figure 3(b), KLF5 knock-down increased ZEB2 protein expression in the context of p53 knock-down, while KLF5 overexpression decreased ZEB2 protein expression in both the p53 wild type cell lines HepG2 and BEL-7402 after p53 was knocked down. Thus, whether KLF5 inhibits EMT or not depends on the p53 status, and these context-dependent effects of KLF5 are likely modulated by ZEB2.

### miR-192 may mediate the p53-dependent regulation of ZEB2 by KLF5

**Figure 4.**
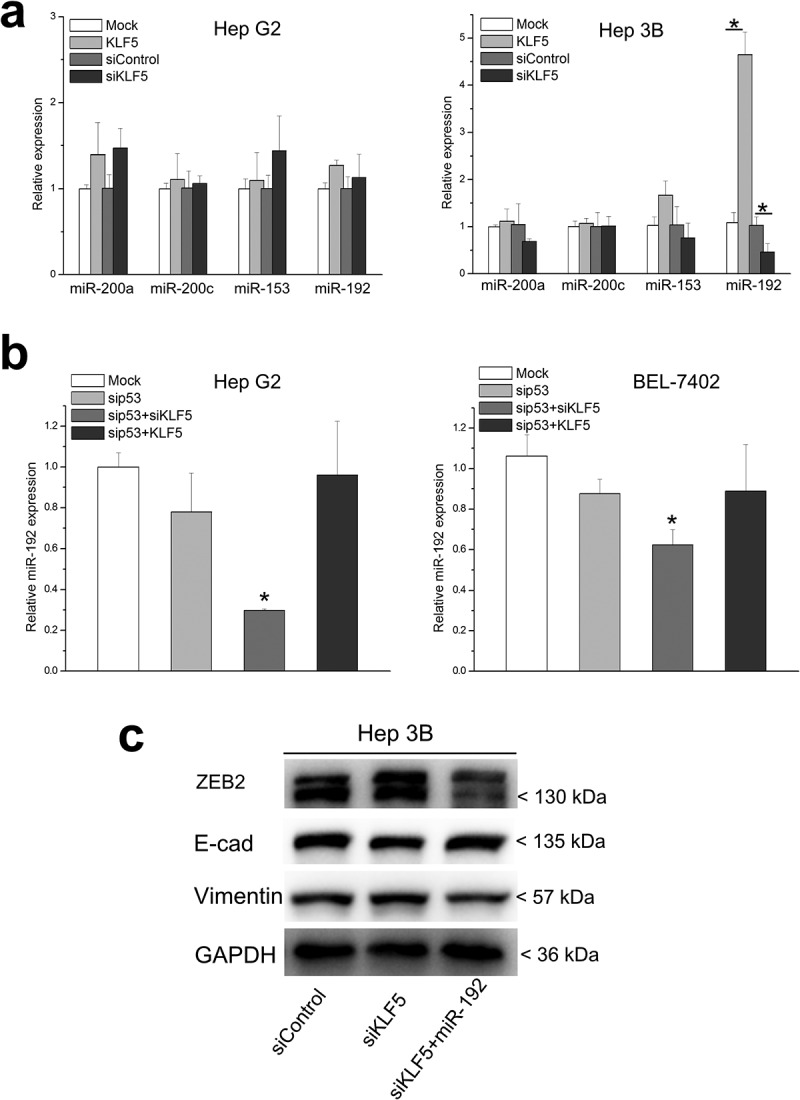
miR-192 mediates the p53-dependent regulation of ZEB1 by KLF5. (a). The expression levels of miRNAs that are induced by p53 and that target ZEB2, including miR-200a, miR-200 c, miR-153 and miR-192, were examined in HepG2 and Hep3B after KLF5 overexpression and knock-down. Only the expression of miR-192 was significantly upregulated in KLF5-overexpressing Hep3B cells and was downregulated in KLF5 knock-down Hep3B cells; the expression of other miRNAs was not significantly different in either HepG2 or Hep3B cells after KLF5 overexpression and knock-down. (b). The expression of miR-192 was significantly decreased in KLF5 knock-down HepG2 (left) and Hep3B (right) cells in the context of p53 silencing. (c). The regulation of ZEB2 and EMT markers, including E-cadherin and vimentin, by KLF5 after KLF5 knock-down was partly rescued by transfection of miR-192 mimics into Hep3B cells. *P < 0.05, data are presented as the means ± SD.

Next, we investigated the mechanism by which p53-dependent KLF5 regulates ZEB2. The mRNA levels of ZEB2 were not significantly altered by changes in p53 and/or KLF5, and only ZEB2 protein levels were obviously different after changes in p53 and/or KLF5 expression. These findings suggest that ZEB2 is regulated by p53 and KLF5 at the posttranscriptional level, such as at the translational or protein (posttranslational) level. Actually, previous studies have reported that p53 could regulate ZEB2 at the translational level through miRNAs, which suggests its involvement in EMT. Therefore, we screened several p53-induced miRNAs that target ZEB2, including miR-200a, miR-200 c, miR-153 and miR-192, in HepG2 and Hep3B cells after KLF5 overexpression and knock-down (Figure 4(a)). Interestingly, none of the miRNAs were significantly altered in HepG2 cells after KLF5 overexpression or knock-down. However, the expression level of miR-192 was significantly upregulated in KLF5-overexpressing Hep3B cells and was downregulated in KLF5 knock-down Hep3B cells, while the other miRNAs did not exhibit any significant expression differences in Hep3B cells after KLF5 overexpression or knock-down. Therefore, we speculate that miR-192 might mediate the p53-dependent regulation of EMT by KLF5, specifically, by regulating the protein expression of ZEB2. To test this, we first assessed the expression of miR-192 in HepG2 and BEL-7402 cells after p53 knock-down and KLF5 overexpression or KLF5 knock-down alone. As shown in Figure 4(b), KLF5 knock-down decreased the expression of miR-192 in HepG2 and BEL-7402 cells after p53 silencing. Those results indicated that the regulatory function of KLF5 on miR-192 expression was dependent on the p53 status. Next, we investigated whether miR-192 mediated the regulation of ZEB2 by KLF5 in the context of p53 knock-down. We forced thee expression of miR-192 mimics in Hep3B knock-down cells and then examined the expression of EMT markers and ZEB2. As shown in Figure 4(c), forced expression of miR-192 mimics attenuated the effect of KLF5 silencing on the expression of E-cadherin, vimentin and ZEB2. Those results suggested that the p53-dependent regulation of ZEB2 by KLF5 possibly occurred via miR-192.

### KLF5 and p53 bind and coordinately regulate miR-192

**Figure 5.**
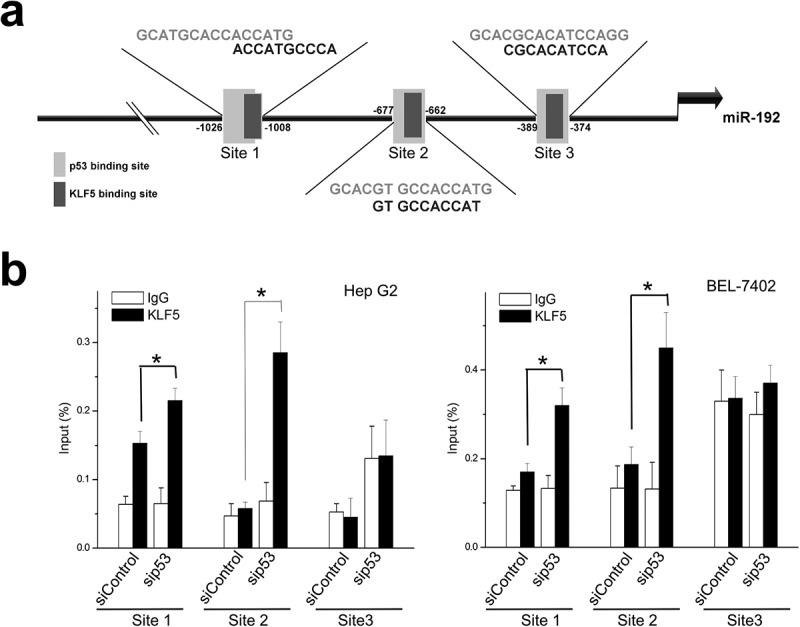
KLF5 directly binds and trans-activates miR-192 in the context of p53 silencing. (a). The 5ʹ upstream regulatory region of miR-192 contains three putative KLF5 binding sites within putative p53 binding sites. (b). Quantitative ChIP revealed that KLF5 binding to the upstream region of miR-192 was markedly increased in HepG2 (left) and BEL-7402 (right) cells in which p53 was silenced compared with controls. *P < 0.05, data are presented as the means ± SD.

We next sought to explore the mechanisms by which p53 status changes caused differential effects of KLF5 on miR-192 expression. We noted that the 5ʹ regulatory region of miR-192 contains three putative KLF5-binding sites that overlap with the p53 response element (Figure 5(a)). We speculated that KLF5 might bind to this region and that this binding might be dependent on p53 status. Actually, using chromatin immunoprecipitation (ChIP), we observed a reciprocal increase in KLF5 binding to miR-192 after p53 knock-down in HepG2 and BEL-7402 cells (Figure 5(b)). Thus, the regulation of miR-192 by KLF5 is dependent on the p53 status, and the function of KLF5 in EMT through miR-192 occurs only in cases of p53 loss.

### In the context of p53 loss or mutation, KLF5 is absent in invasive liver cancer

**Figure 6.**
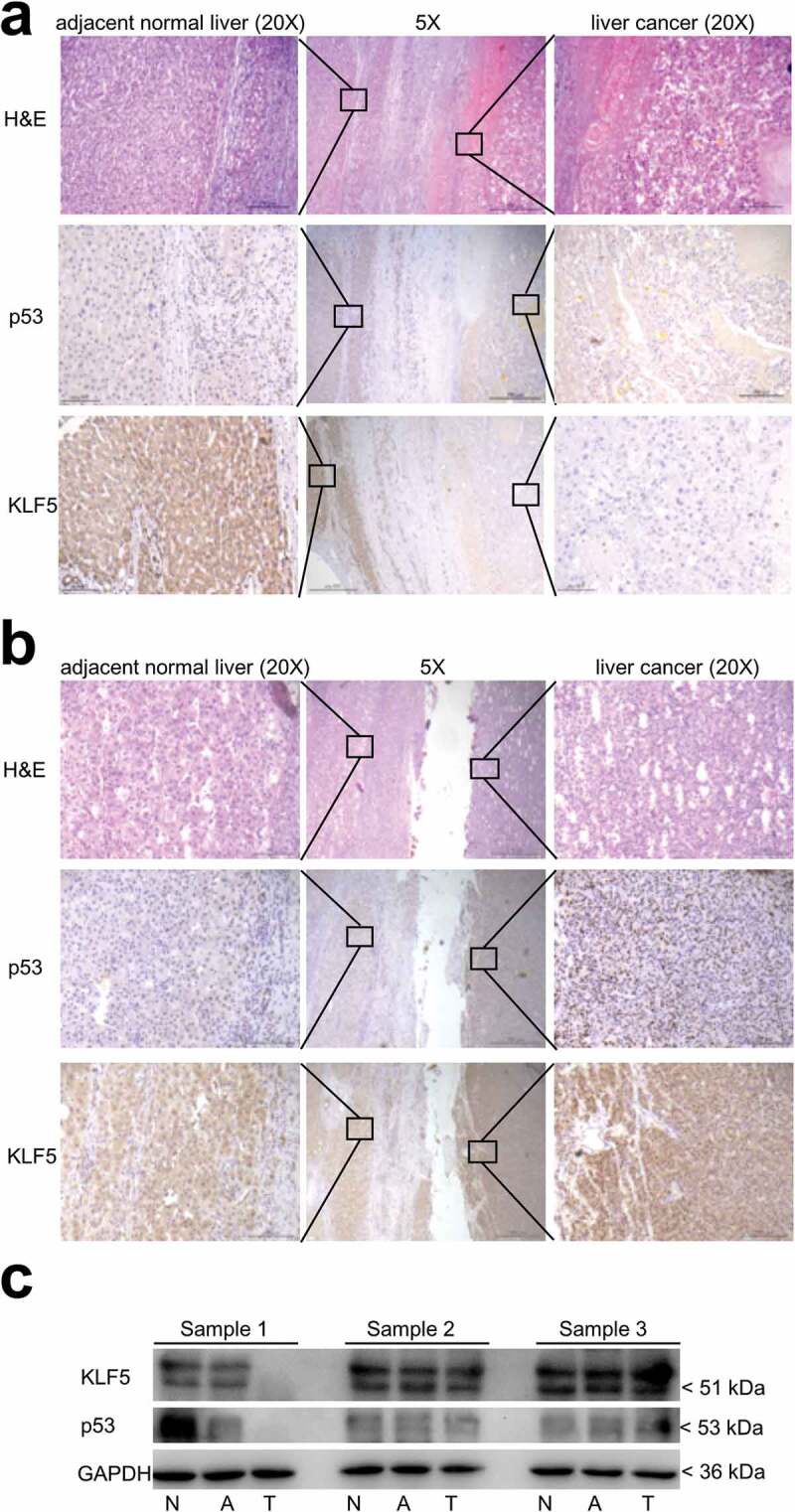
Expression of KLF5 is lost in invasive liver cancer. (a). In liver cancer with metastasis, p53 and KLF5 were both lost in the tumor tissue compared with the adjacent normal liver tissue. (b). In liver cancer without metastasis, although p53 was mutated in the tumor tissue, the expression of KLF5 was still positive in the tumor tissue compared with the adjacent normal liver tissue. H&E (hematoxylin and eosin) staining was used to identify the tumor tissue and adjacent normal liver tissue. Scale bars = 500 µm (5×) and 100 µm (20×). (c). The expression levels of KLF5 and p53 in three liver cancer samples (sample 1, sample 2 and sample 3) were examined by western blotting. In a sample with metastasis, KLF5 and p53 were both lost in the tumor tissue (T) compared with normal (N) and adjacent normal liver tissue (A), while in the samples without metastasis, the expression of both KLF5 and p53 was detected.

The results discussed above clearly indicate that KLF5 could compensate for the functions of p53 in EMT and migration inhibition in liver cancer cells. Thus, KLF5 loss might be a critical event in human liver cancer invasion in the context of p53 loss or mutation. Therefore, we used IHC to examine KLF5 expression in liver cancers with and without metastasis in which p53 was lost or mutated. Interestingly, as shown in Figure 6(a), in a case of liver cancer with metastasis, the adjacent normal liver tissue expressed p53 and KLF5, while p53 and KLF5 expression was lost in the tumor tissue. However, in a case of liver cancer without metastasis (Figure 6(b)), although p53 was mutated in the tumor tissue (elevated and stable protein expression of mutated p53), KLF5 was still positively expressed in the tumor tissue. Furthermore, we used western blot to examine KLF5 and p53 expression in another three liver cancer samples with and without metastasis. In another case of liver cancer with metastasis, p53 expression was lost in the tumor tissue (T), and importantly, KLF5 expression was also lost relative to normal (N) and adjacent normal liver tissue (A). On the contrary, in other cases of liver cancer without metastasis, the expression of KLF5 and p53 was detected by western blot (Figure 6(c)). Those results suggested that, in the context of p53 loss or mutation, KLF5 loss could be a valuable diagnostic target for invasive liver cancer.

## Discussion

Here, we demonstrate that the regulation of liver cancer cell epithelial-mesenchymal transition (EMT) by KLF5 is context-dependent, and specifically, that the function of KLF5 in liver cancer cell EMT requires p53 mutation or loss.

Although many studies have reported the function of KLF5 in cell migration and even EMT, opposing results (promotion or suppression) have been reported in different studies. Specifically, KLF5 promotes the migration of bladder cancer cells [7,11], breast cancer cells [10,20], mouse primary esophageal keratinocytes [9], bronchial smooth muscle cells [21] and intestinal epithelial cells [22]; in contrast, KLF5 inhibits the migration of HaCaT cells, MCF-10A cells [23] and mouse PDA cells [24]. Notably, an earlier study revealed that, after KLF5 knock-down, cell migration could occur and EMT-related genes could be expressed only if p53 was mutated or if its expression was ablated [12]. We also observed that the effect of KLF5 on liver cancer cell migration and EMT was dependent on the silencing or loss of p53, which was consistent with the results of previous studies.

Most importantly, we explored the mechanisms by which KLF5 and p53 coordinately regulate EMT. EMT is a complicated but critical cellular process by which epithelial cells lose their epithelial characteristics and acquire a mesenchymal-like phenotype [15]. In addition to the loss of cell-cell adhesion, which is mediated by E-cadherin downregulation and the upregulation of mesenchymal markers such as vimentin, EMT activators (transcription factors (TFs)) such as Snail, Slug, Twist, ZEB1, and ZEB2 also play central roles in EMT [14,16,17]. Actually, several reports have shown the transcriptional activation of EMT activators by p53 and KLF5. Specifically, ZEB1 and ZEB2 have been identified as the primary EMT regulators of p53 [25], and on the contrary, KLF5 has been found to suppress EMT in HaCaT cells via ZEB1, whereas the expression of ZEB2 was not detected in HaCaT cells [23]. ZEB2 is the mammalian paralog of ZEB1, and they both act as transcriptional repressors by binding to the E box (5ʹ-CANNTG-3ʹ) of the E-cadherin promoter to induce EMT [26]. However, the regulation of ZEB1 and ZEB2 by p53 and KLF5 is indirect, as miRNAs mediate the effects of p53 and KLF5 on the expression of ZEB1 and ZEB2, and thus EMT [23,25,27,28]. Consistent with those previous studies, we also found that the mRNA expression levels of ZEB1 and ZEB2 were not changed after p53 and KLF5 expression was altered, and only the expression of ZEB2 protein was obviously changed. Therefore, miRNAs might mediate the effects of p53 and KLF5 on ZEB2.

miRNAs are noncoding small RNAs that usually silence or repress gene expression by targeting the 3ʹ untranslated regions (UTRs) of mRNAs. Increasing numbers of miRNAs have been identified as negative regulators of EMT [29,30], and many of the miRNAs engaged in targeting EMT-TFs are transcriptionally activated by p53 [27,28,31]. For example, p53 can regulate EMT through the targeting of ZEB1 by miR-200s and the targeting of ZEB2 by miR-192 [25,32,33]. Interestingly, several recent studies have also revealed the role of KLF5 in EMT through its regulation of miRNAs [23]. In this study, we clearly demonstrate that KLF5 participates in the transcriptional regulation of miR-192, which is also a miRNA induced by p53. Furthermore, we observed reciprocal binding of KLF5 and p53 to the 5ʹ regulatory region of miR-192 and that p53 preferentially binds to miR-192; however, in cells in which p53 is suppressed, KLF5 was bound to and transcribed miR-192. Actually, this reciprocal binding relationship was similar to that which was previously observed for Notch1 [12] and p21Waf1/Cip1 [5], and the expression of Notch1 and p21Waf1/Cip1 has been found to be dependent on p53 status. However, although miRNA-200s has been reported to be regulated by p53 and KLF5, we found that the p53 and KLF5 binding sites at the 5ʹ regulatory region of miR-200s were different, and therefore, the regulation of miR-200s by p53 and KLF5 is not coordinate. In addition, the target of miR-200s is usually ZEB1, and we did not observe any significant change in ZEB1 expression after KLF5 was altered in p53 wild type and p53-null liver cancer cells. Thus, our study reveals a novel mechanism of miR-192 involvement in the p53-dependent regulation of KLF5 in liver cancer cell EMT.

The loss of p53 activity has been described in many types of human tumors, including in 30%-60% of HCC [34,35]. Almost 80% of p53 mutations in cancer are missense, which results in the synthesis of a stable protein that lacks typical DNA binding activity [36]. Here, we identify KLF5 as a key determinant of invasive liver cancer. Invasive liver cancer usually exhibits KLF5 loss together with a naturally occurring p53 loss or mutation. Consistently, in invasive esophageal squamous carcinomas in which p53 is mutated or lost early in esophageal tumorigenesis, KLF5 expression is markedly decreased [12]. Thus, KLF5 loss combined with p53 loss or mutation might be a valuable diagnostic target in invasive cancer.

**Figure 7.**
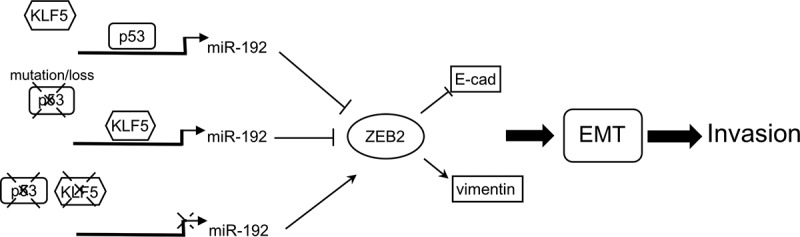
Model for KLF5 and p53 regulation of miR-192 and their involvement in liver cancer cell EMT.

In conclusion, we propose a model (Figure 7) in which p53 normally binds to miR-192 but where KLF5 can also bind to miR-192 when p53 binding is lost (as a result of p53 loss or mutation), which then transactivates miR-192 in place of p53 to suppress liver cancer cell EMT. However, when KLF5 is lost, miR-192 transcription is inactivated. The protein level of ZEB2, a target of miR-192 and an activator of EMT, increases and EMT and invasion are promoted. Thus, our findings explain the mechanisms of the p53-dependent effect of KLF5 on liver cancer cell EMT and suggest that KLF5 loss is a valuable diagnostic and therapeutic target for invasive liver cancer, and potentially for other cancers associated with p53 loss or mutation.

## Materials and methods

### Cell lines and human liver samples

The human liver cancer cell lines HepG2, BEL-7402 and Hep3B were cultured in Eagle’s Minimum Essential Medium (MEM, Sigma, CA, USA) supplemented with 10% fetal calf serum (FCS, Sigma, USA), 100 U/mL penicillin, and 100 mg/mL streptomycin and incubated at 37°C with 5% CO_2_.

All studies involving human tissues were evaluated by the local ethics committee (Beijing Friendship Hospital, Capital Medical University, Beijing, China), and all samples were collected from subjects who provided informed consent for his/her tissue to be used for research purposes. Samples that contained adjacent normal (A) and liver tumor (T) tissue that were used for immunochemistry (n = 10) were obtained as paraffin blocks and were sectioned at a thickness of 4 µm. A representative sample was stained with hematoxylin and eosin and evaluated for the presence of normal liver and tumor tissue. Tissues that contained normal (N), adjacent normal (A) and liver tumor (T) that were used for western blotting (n = 3) were obtained as frozen samples. The characteristics of patients are listed in Table S1.

### Antibodies and reagents

Anti-ZEB1 and anti-GAPDH antibodies were purchased from Santa Cruz Biotechnology (CA, USA). The anti-E-cadherin, anti-vimentin, anti-snail, anti-slug, anti-twist and anti-p53 antibodies were purchased from Cell Signaling Technology (MA, USA). The anti-KLF5 antibody was purchased from Thermo Fisher (CA, USA). The anti-ZEB2 antibody was purchased from Proteintech (Rosemont, USA). Horseradish peroxidase (HRP)-conjugated goat anti-rabbit and goat anti-mouse secondary antibodies were purchased from Santa Cruz Biotechnology (USA). KLF5 siRNA (siRNA ID stQ0005721-1), p53 siRNA (siRNA ID stB0002017C-1-5) and control siRNA were purchased from RiboBio (Guangzhou, China) and were used according to the manufacturer’s instructions.

### Generation of cell lines expressing KLF5

When the cells were 80% confluent, they were transfected with hU6-MCS-CMV-3FLAG-SV40-Neomycin/human KLF5 (KLF5) or empty plasmids (mock) by Lipofectamine 3000 (Invitrogen, CA, USA) according to the manufacturer’s instructions. The culture medium was changed 6 h after transfection. For different assays, cells were harvested 24 h or 48 h after transfection.

### Real-time PCR

Total RNA was isolated using TRIzol reagent (Invitrogen, USA). For regular real-time PCR, 2 μg of RNA was reverse-transcribed in 20 μL of reaction buffer using a reverse transcriptase kit (Roche, USA). The primers used are listed in Table S2; GAPDH was used as an internal control. For miRNA real-time PCR, 1 μg of miRNA was reverse-transcribed using a miRcute Plus miRNA First-Strand cDNA Synthesis Kit (TIANGEN, China). The primers for miR-200b, miR-200 c, miR-153 and miR-192 were purchased from TIANGEN (Beijing, China), and miRNA real-time PCR was performed using a miRcute Plus miRNA qPCR Detection Kit (TIANGEN, China) according to the manufacturer’s instructions. For real-time PCR, thermal cycling conditions were as follows: 95°C for 10 min, followed by 40 cycles of 95°C for 30 sec and 60°C for 1 min.

### Western blotting

Cells were lysed in lysis buffer (50 mM Tris-HCl pH 8, 150 mM NaCl, 5 mM ethylenediaminetetraacetic acid (EDTA), 1% NP40, a protease inhibitor cocktail (Roche, USA) and phosphatase inhibitors (Roche, USA)). Proteins were separated and transferred onto PVDF membranes (Amersham Biosciences) using a Bio-Rad wet transfer unit. After blocking with 5% (w/v) nonfat dried milk in TBST solution (0.05% (v/v) Tween-20) for 1 h at room temperature, the membranes were incubated with primary antibody overnight at 4°C, which was followed by incubation with a horseradish peroxidase (HRP)-conjugated secondary antibody (1:5000) for 1 h at 37°C. The bands were then detected using Immobilon Western Chemiluminescent HRP Substrate (Millipore, USA).

### Immunofluorescence staining

Cells were fixed in 4% paraformaldehyde in PBS for 15 min and then permeabilized in 0.3% Triton X-100 for 10 min. Nonspecific binding sites were blocked by incubation in 5% bovine serum albumin for 15 min. After they were washed in PBS, the cells were incubated with anti-E-cadherin and anti-vimentin (1:200) overnight at 4°C. After additional washes in PBS, the cells were incubated with a mixture of anti-rabbit AlexaFluor 488-conjugated secondary antibodies (1:200) and Rhodamine-conjugated phalloidin at 5 U/mL (Molecular Probes, USA) for 2 h at room temperature. Following washes in PBS, the cells were mounted on a slide in mounting medium containing DAPI (Molecular Probes, USA). The cells were examined and imaged with a confocal microscope (FV 300, Olympus).

### Transwell migration assay

For the migration assay, Boyden chambers with filter inserts (pore size, 8 μm) were used. In the upper chamber, 1 × 10^5^ cells in 200 μL of MEM medium were seeded, while 1.5 mL of complete MEM was added to the lower chamber. After 12–36 h, the cells were fixed and stained with 0.05% crystal violet in PBS for 15 min. After the cells on the upper side of the filters were removed by cotton-tipped swabs, the cells on the underside of the filters were counted under a microscope.

### Immunohistochemistry

Tissues were fixed in 4% paraformaldehyde in PBS and then embedded in paraffin. After the slides were deparaffinized in xylene and rehydrated in alcohol, the sections were subjected to antigen retrieval by heating them in a microwave in sodium citrate buffer for 15 min. To block endogenous peroxidase activity, the sections were treated with 0.3% hydrogen peroxide for 15 min and then further treated with FBS to block nonspecific binding sites. Sections were incubated with a primary antibody against p53 (1:200) or KLF5 (1:500) overnight at 4°C. After washes in PBS, the sections were incubated with the appropriate biotinylated secondary antibody for 1 h at room temperature after which the protein expression was visualized by 3,3ʹ-diaminobenzidine tetrahydrochloride (DAB) staining. Sections were viewed and images were obtained using a microscope.

### ChIP assay

HepG2 or BEL-7402 cells were transfected with p53 siRNA or control siRNA using Lipofectamine 3000 reagent (Invitrogen, USA). Twenty-four hours after transfection, the cells were harvested, and a ChIP assay was performed using a chromatin immunoprecipitation (ChIP) assay kit (Cell Signaling Technology, USA). Precipitated DNA was subjected to real-time PCR with miR-192 promoter primers (Table S2).

### Statistical analysis

SPSS software version 18.0 (SPSS INC., Chicago, IL, USA) was used to perform all statistical analyses. The data are presented as the means ± standard deviation (SD). Unless stated otherwise, the statistical analyses of the results from all experiments were nonparametric statistics. Specifically, the paired data were analyzed by Wilcoxon-signed rank test, while the unpaired data were analyzed by Mann-Whitney test. Significance was considered when *P* values were less than 0.05.

## Supplementary Material

Supplemental Material
