# Supplementary material for: KLF5 regulates epithelial-mesenchymal transition of liver cancer cells in the context of p53 loss through miR-192 targeting of ZEB2
Source: Cell Adh Migr. 2020 Oct 7;14(1):182–94. doi: 10.1080/19336918.2020.1826216 (PMC7553557; doi:10.1080/19336918.2020.1826216)

**Supplementary Data**

**1. Supplementary Figure and Legend**


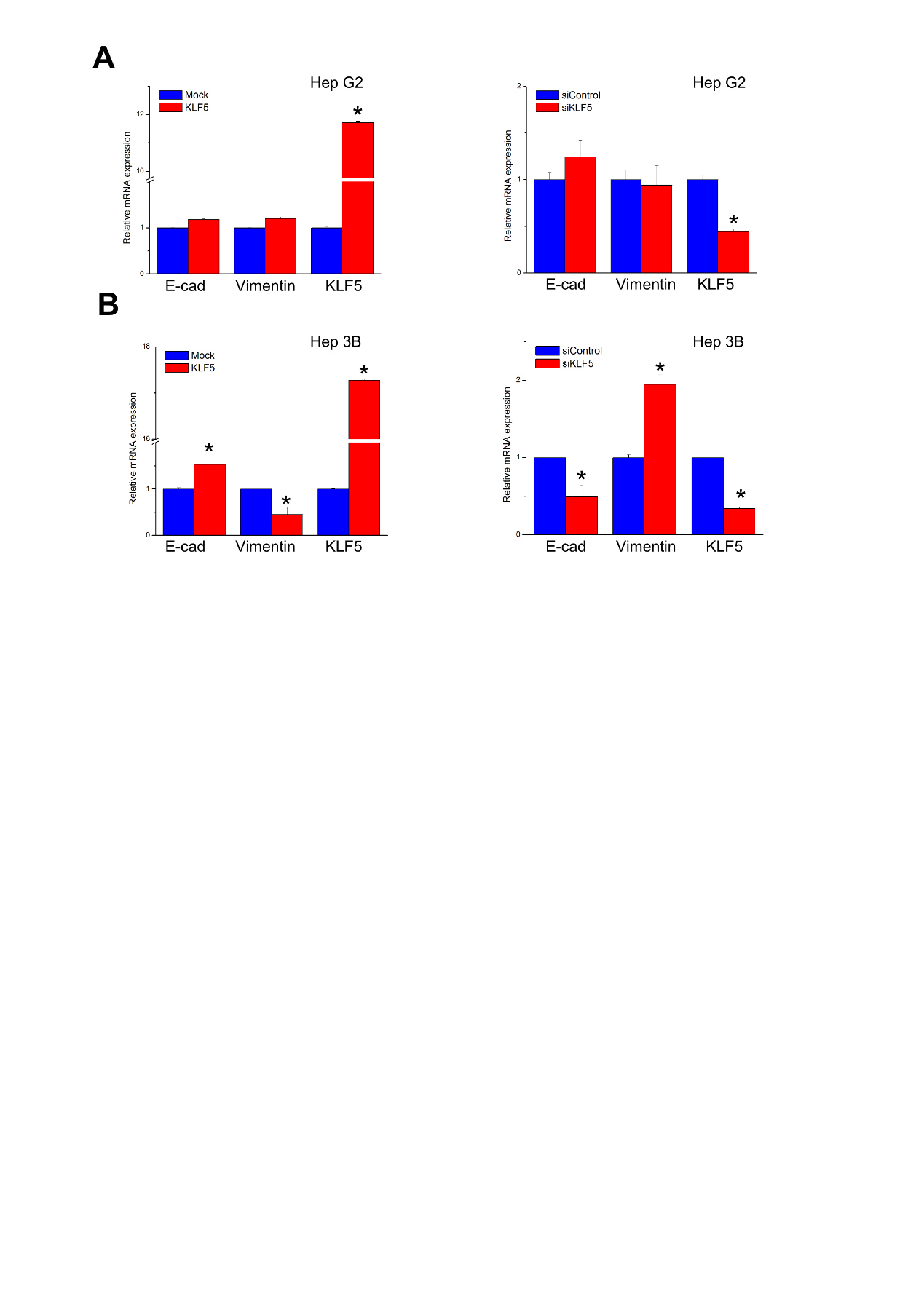


Figure S1. A. The mRNA expressions of E-cadherin and vimentin did not alter after KLF5 over-expression or knock-down in p53 wild-type Hep G2 cells. B. In p53 null Hep 3B cells, the mRNA expression of E-cadherin increased and the mRNA expression of vimentin decreased in KLF5 over-expressed cells, and the mRNA expression of E-cadherin decrease and the mRNA expression of vimentin increased in KLF5 knock-down cells. **P*<0.05, data are presented as the means ± SD.


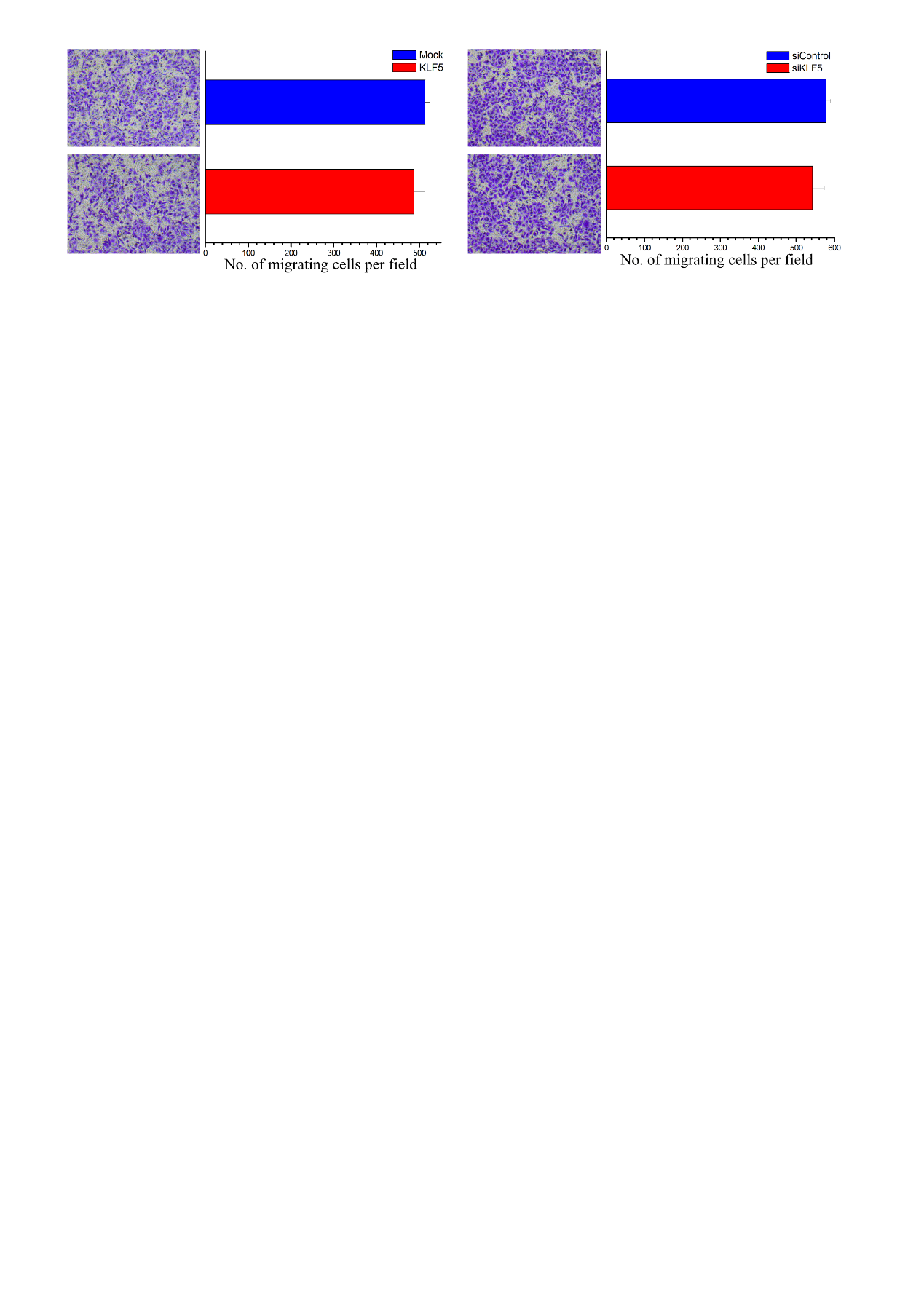


Figure S2. KLF5 did not influence the cell migration of BEL-7402 cells harboring wild type p53.


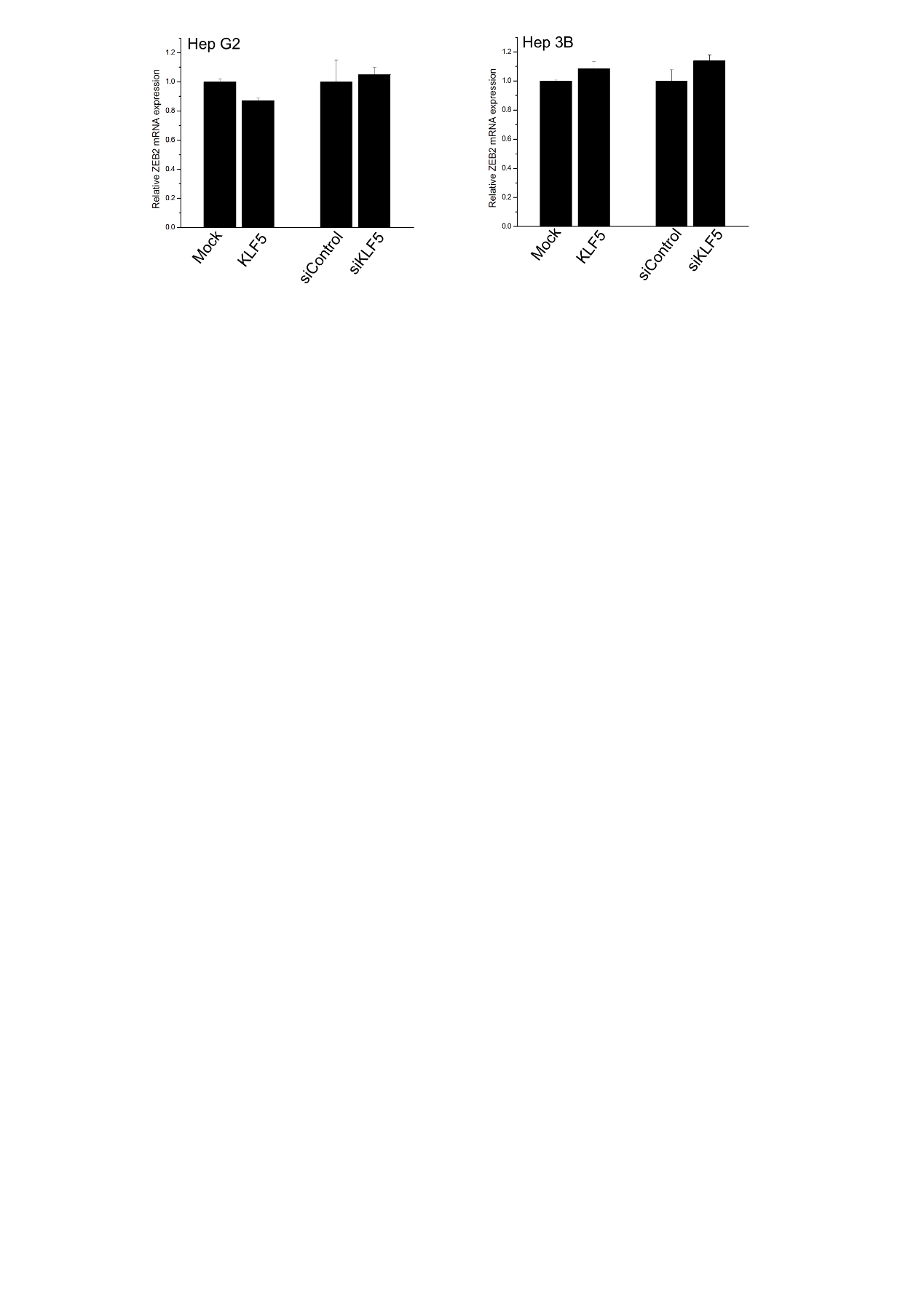


Figure S3. The mRNA expression of ZEB2 did not exhibit significant changes in Hep G2 and Hep 3B cells after KLF5 over-expresssion or knock-down.

**2. Supplementary Table**

**Table S1.** The characteristics of liver cancer patients

| **Gender** | **Age** | **p53 expression** | **KLF5 expression** | **Metastasis** | **Application** |
| --- | --- | --- | --- | --- | --- |
|  |  | **(T compared to A)** | **(T compared to A)** | **(N/Y)** |  |
| Male | 52 | + | = | N | IHC |
| Male | 44 | = | + | N | IHC |
| Female | 51 | + | - | Y | IHC |
| Male | 57 | - | - | Y | IHC |
| Male | 42 | - | - | Y | IHC |
| Female | 73 | + | = | N | IHC |
| Female | 65 | + | + | N | IHC |
| Male | 56 | - | - | Y | IHC |
| Male | 62 | + | = | N | IHC |
| Male | 60 | + | + | N | IHC |
| Male | 61 | - | - | Y | WB (sample 1) |
| Female | 47 | = | - | N | WB (sample 2) |
| Male | 37 | + | + | N | WB (sample 3) |

+:the expression of p53/KLF5 in cancer tissue (T) is higher than adjacent normal liver tissue (A); =: the expression of p53/KLF5 in cancer tissue (T) is similar to adjacent normal liver tissue (A); -: the expression of p53/KLF5 in cancer tissue (T) is lower than adjacent normal liver tissue (A), N: liver cancer without metastasis; Y: liver cancer with metastasis, IHC: Immunohistochemistry, WB: Western blotting.

| **Table S2.** Primers used for PCR | | | | |
| --- | --- | --- | --- | --- |
| Name | Accession No. |  | Sequences (5'-3') | Product Size, bp |
| E-cad | NM_004360.3 | Forward | CGAGAGCTACACGTTCACGG | 119 |
|  |  | Reverse | GGGTGTCGAGGGAAAAATAGG |  |
| vimentin | NM_003380.2 | Forward | AGTCCACTGAGTACCGGAGAC | 98 |
|  |  | Reverse | CATTTCACGCATCTGGCGTTC |  |
| ZEB2 | NM_014795.3 | Forward | CCCTGGCACAACAACGAGAT | 104 |
|  |  | Reverse | ATTGCGGTCTGGATCGTGG |  |
| KLF5 | NM_001730.4 | Forward | ACACCAGACCGCAGCTCCA | 165 |
|  |  | Reverse | TCCATTGCTGCTGTCTGATTTGTAG |  |
| GAPDH | NM_002046.5 | Forward | GAGTCAACGGATTTGGTCGT | 185 |
|  |  | Reverse | GACAAGCTTCCCGTTCTCAG |  |
| miR-192 promoter  Site 1 for ChIP |  | Forward | TCCTGGGTTCAAGCGATTCT | 81 |
|  |  | Reverse | AAATTAGCTGGGCATGGTGG |  |
| miR-192 promoter  Site 2 for ChIP |  | Forward | CACACAGTCCTCCCATCTCA | 101 |
|  |  | Reverse | GTGAAACCCCATCTCTACGAA |  |
| miR-192 promoter  Site 3 for ChIP |  | Forward | ATTGAGCCCTTTCTGTCTGC | 90 |
|  |  | Reverse | CTGTGTCCTCCTGTCCCAAA |  |

**3. The densitometry of immunoblot**

The densitometry of Figure 1


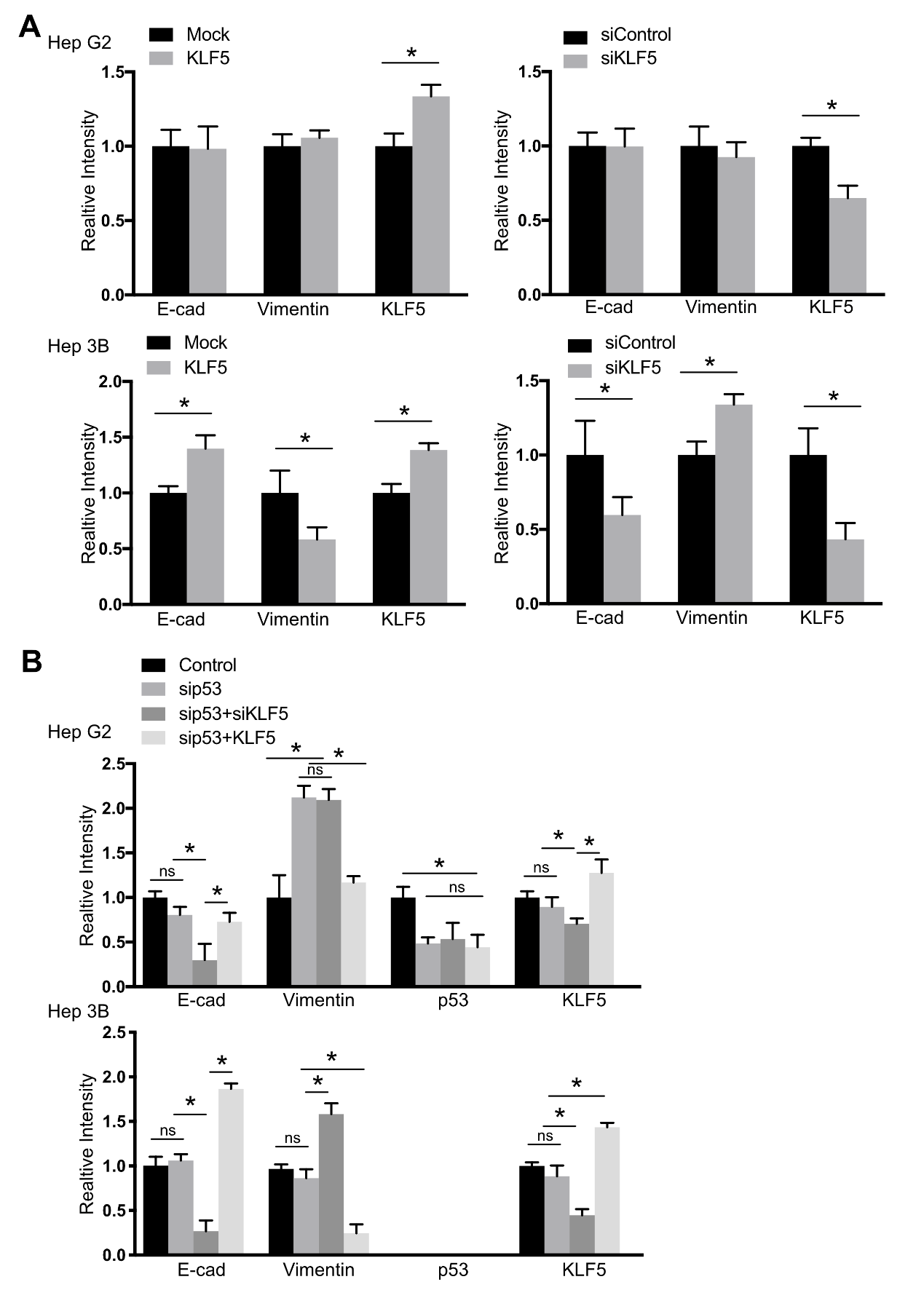


**P*<0.05, ns *P*>0.05, data are presented as the means ± SD.

The densitometry of Figure 3


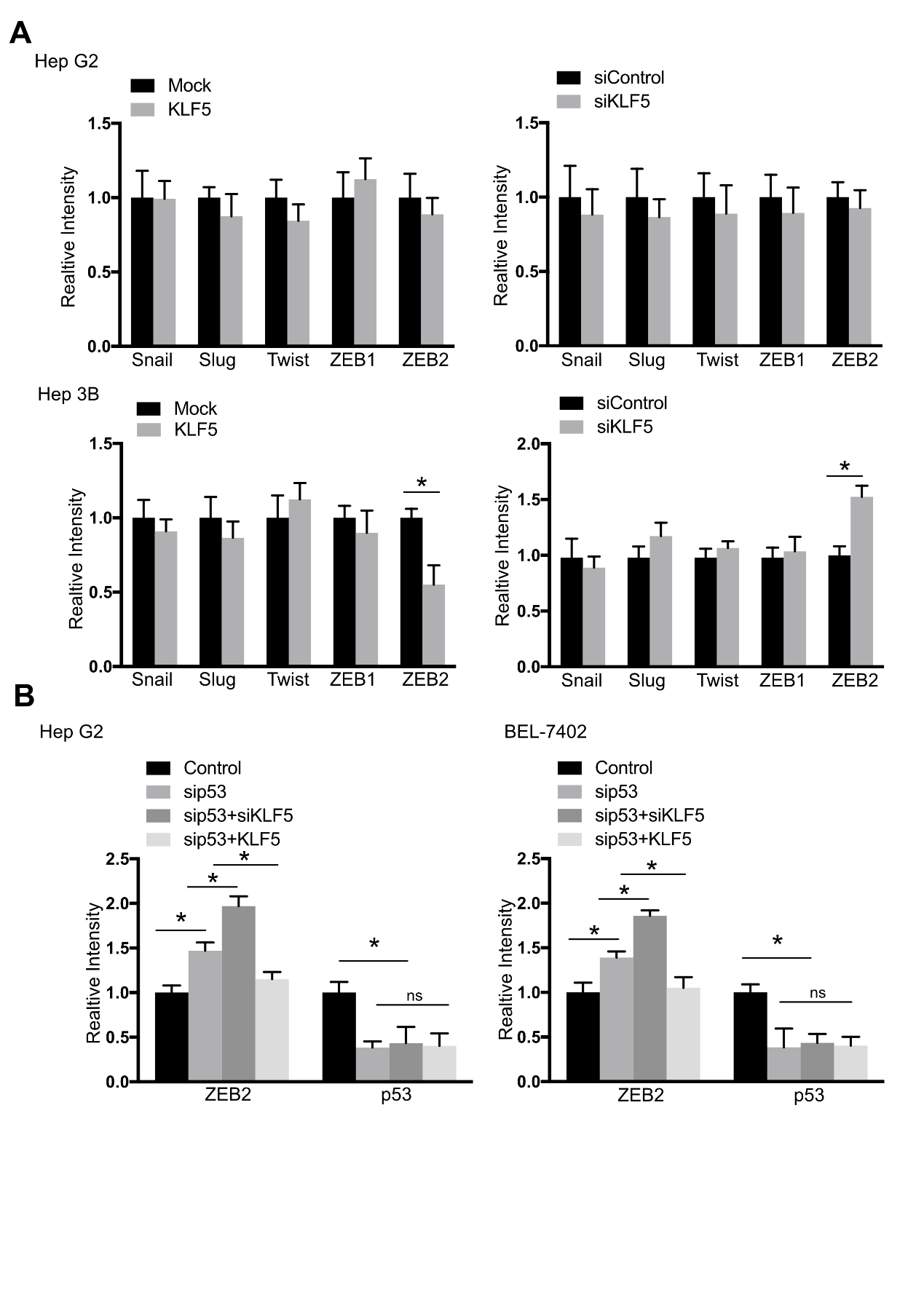


**P*<0.05, ns *P*>0.05, data are presented as the means ± SD.

The densitometry of Figure 4C


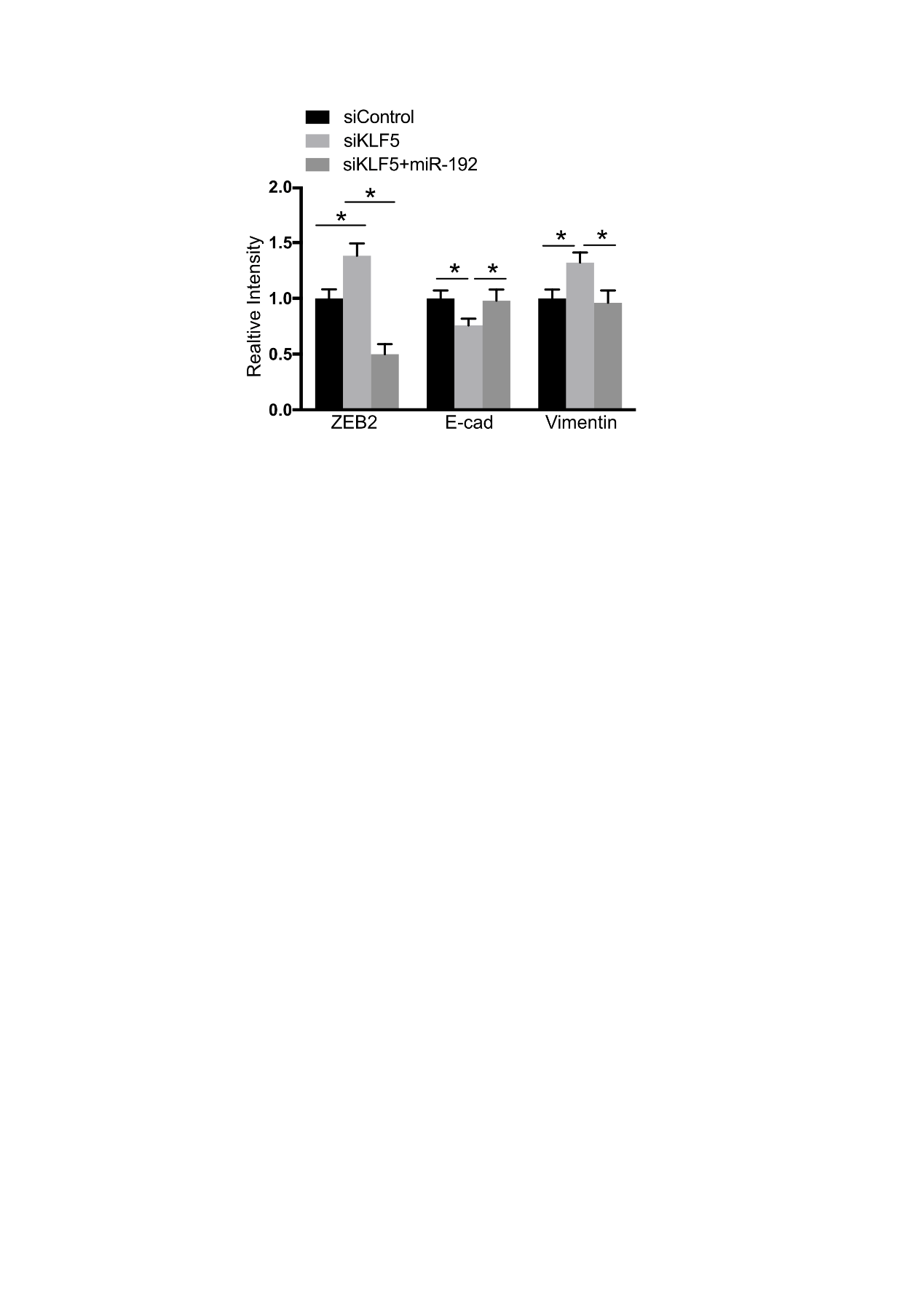


**P*<0.05, data are presented as the means ± SD.

**4. The amplification plot of real time PCR**

The amplification plot of Figure 4

A

Hep G2

miR-200a


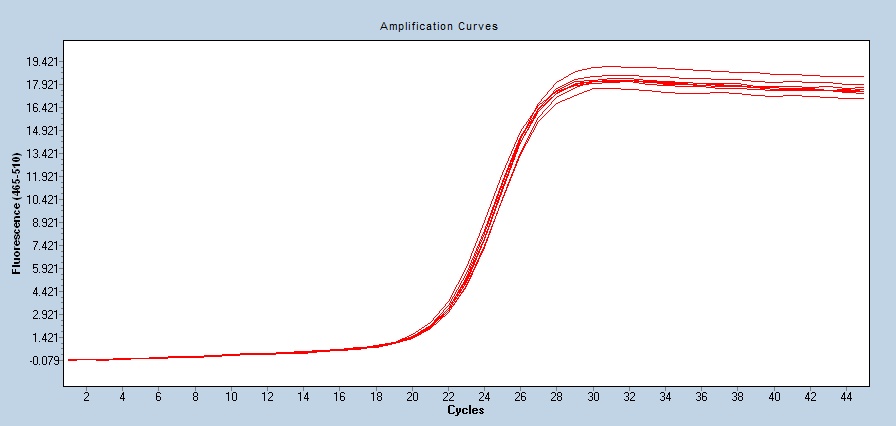


miR-200c


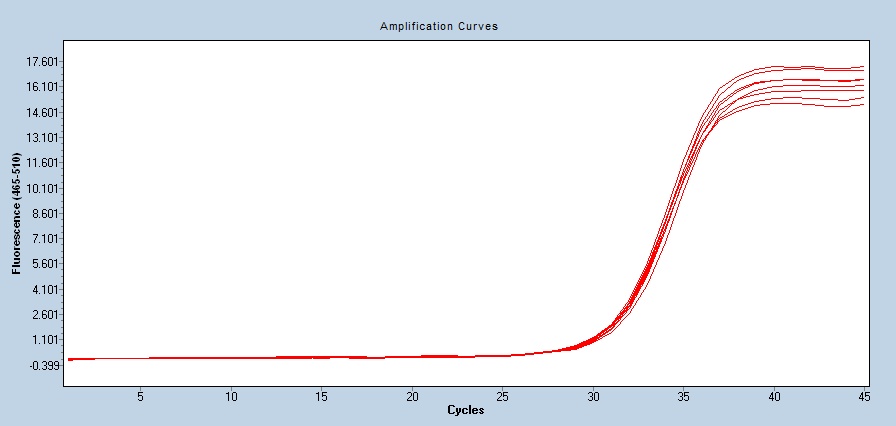


miR-153


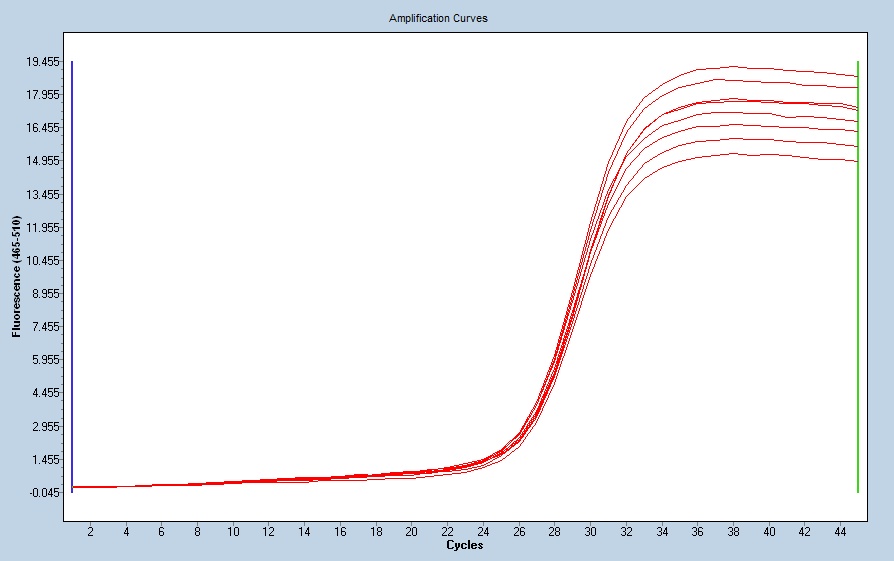


miR-192


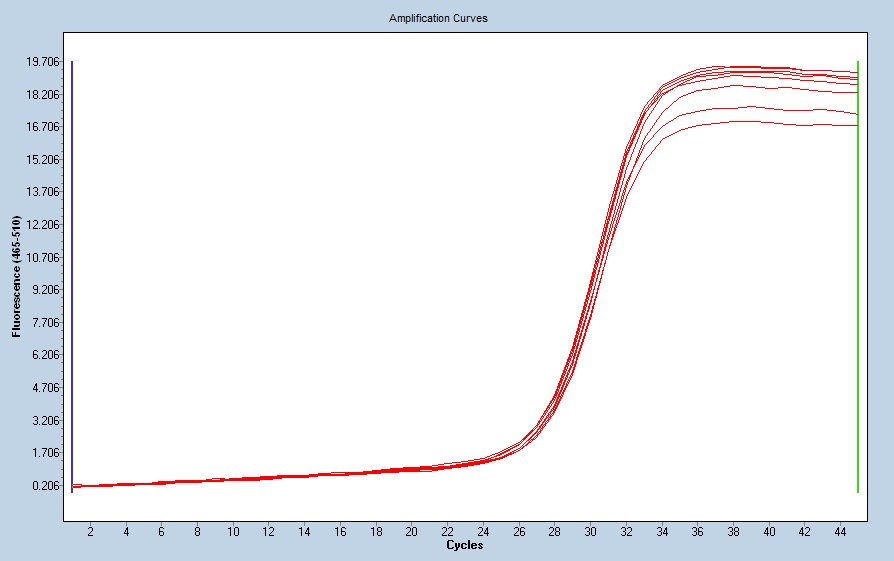


Hep 3B

miR-200a


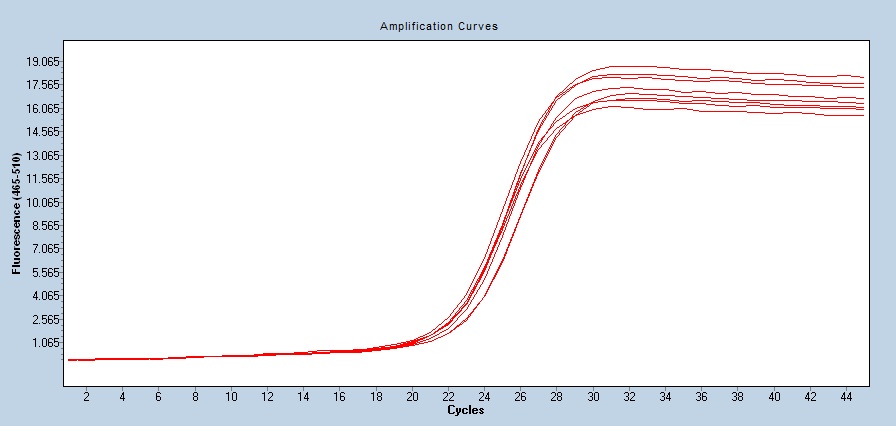


miR-200c


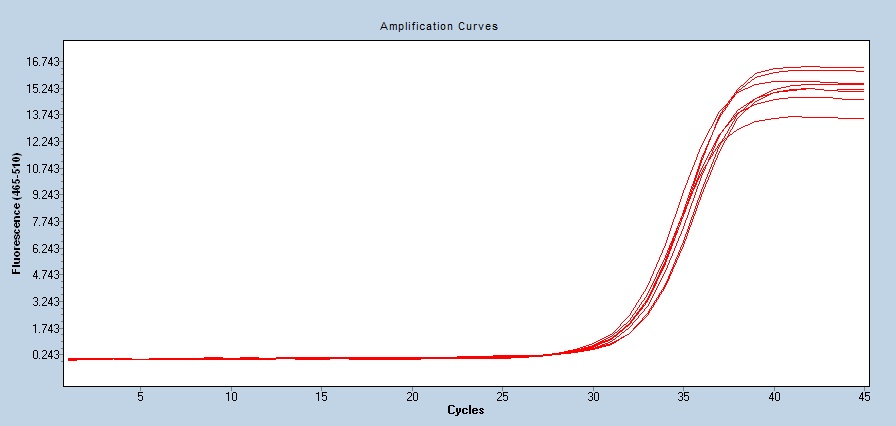


miR-153


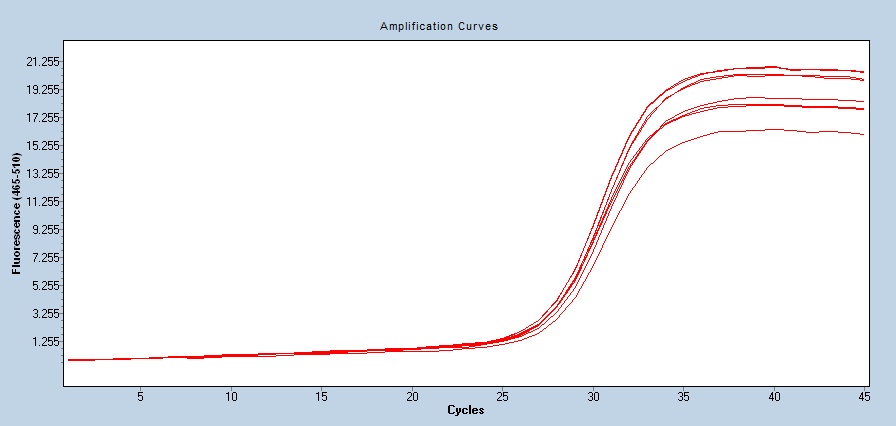


miR-192


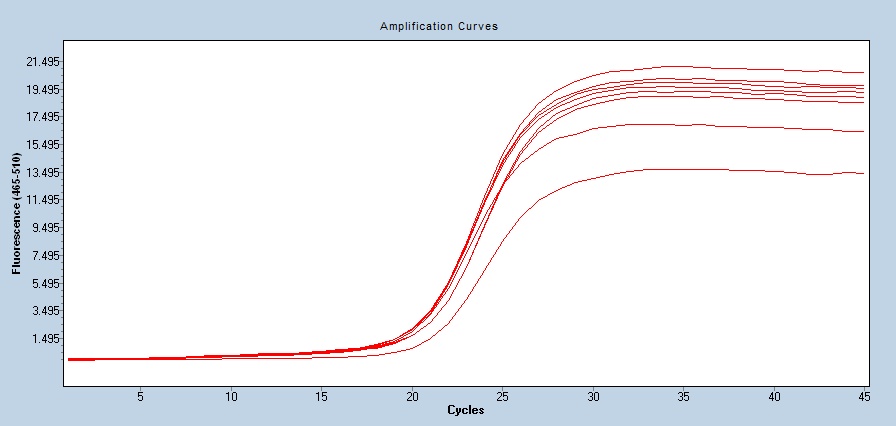


B

Hep G2


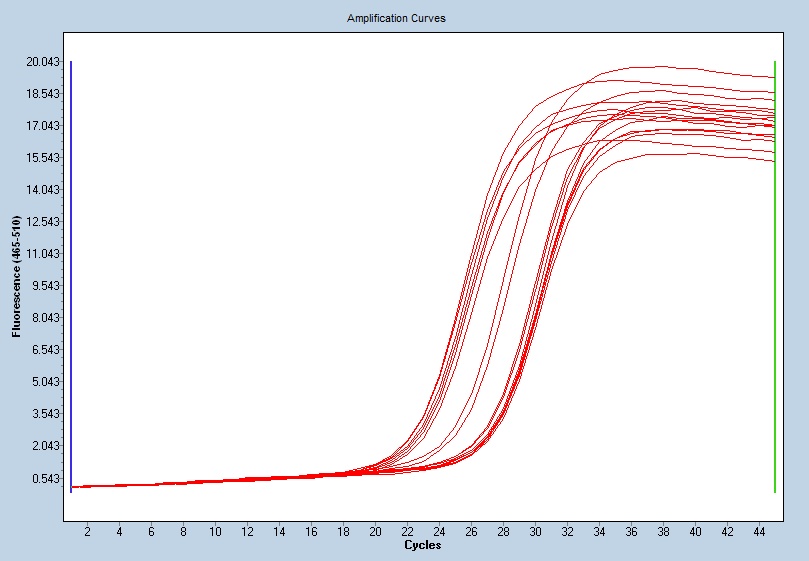


BEL-7402


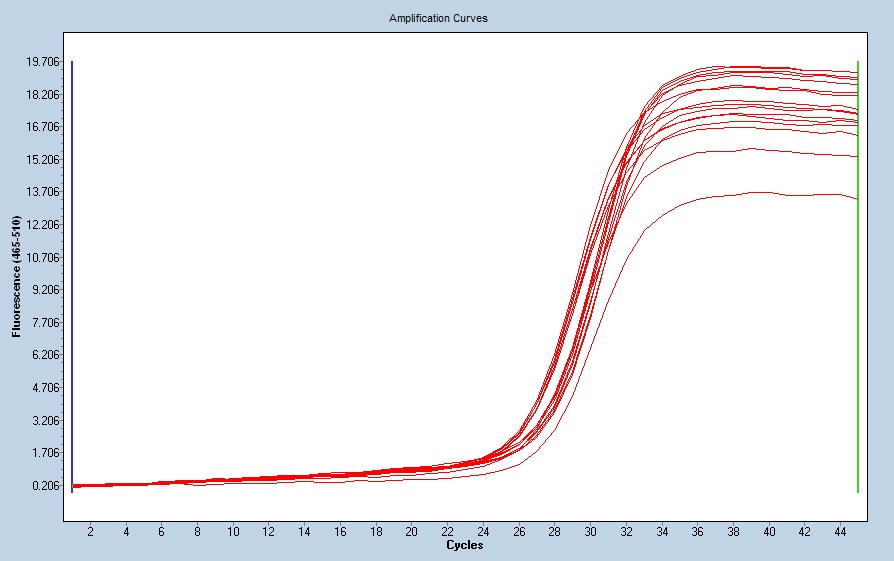


The amplification plot of Figure 5

B

Hep G2


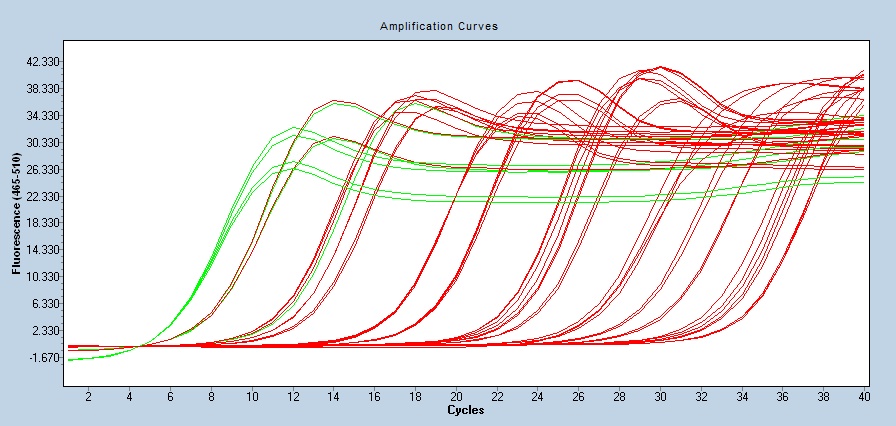


BEL-7402


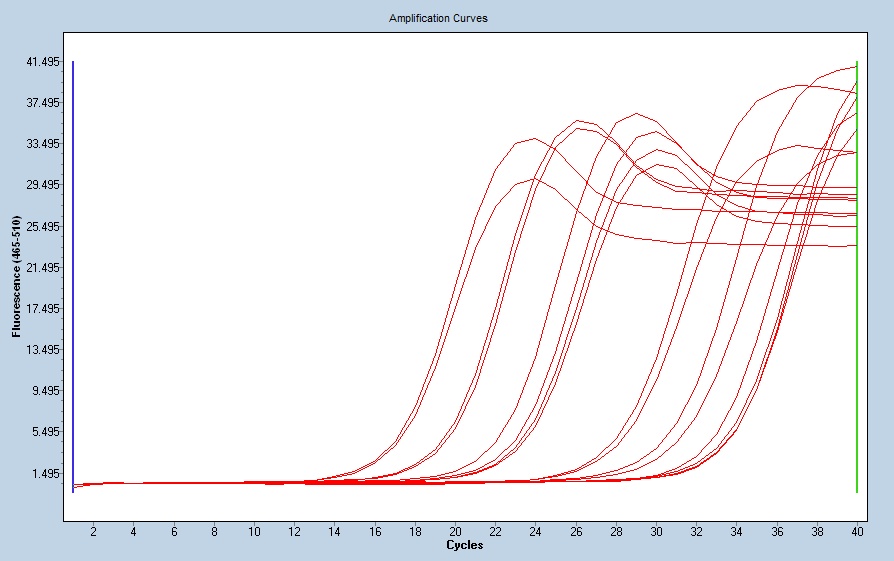

Supplement: Supplemental Material [file KCAM_A_1826216_SM8335.docx]
